# Integrated ZnO Nano-Electron-Emitter with Self-Modulated Parasitic Tunneling Field Effect Transistor at the Surface of the p-Si/ZnO Junction

**DOI:** 10.1038/srep33983

**Published:** 2016-09-22

**Authors:** Tao Cao, Laitang Luo, Yifeng Huang, Bing Ye, Juncong She, Shaozhi Deng, Jun Chen, Ningsheng Xu

**Affiliations:** 1 State Key Laboratory of Optoelectronic Materials and Technologies, Guangdong Province Key Laboratory of Display Material and Technology, School of Electronics and Information Technology, Sun Yat-sen University, Guangzhou 510275, People’s Republic of China; 2 Sun Yat-sen University-Carnegie Mellon University (SYSU-CMU) Shunde International Joint Research Institute, Shunde 528300, People’s Republic of China

## Abstract

The development of high performance nano-electron-emitter arrays with well reliability still proves challenging. Here, we report a featured integrated nano-electron-emitter. The vertically aligned nano-emitter consists of two segments. The top segment is an intrinsically lightly n-type doped ZnO nano-tip, while the bottom segment is a heavily p-type doped Si nano-pillar (denoted as p-Si/ZnO nano-emitter). The anode voltage not only extracted the electron emission from the emitter apex but also induced the inter-band electron tunneling at the surface of the p-Si/ZnO nano-junction. The designed p-Si/ZnO emitter is equivalent to a ZnO nano-tip individually ballasted by a p-Si/ZnO diode and a parasitic tunneling field effect transistor (TFET) at the surface of the p-Si/ZnO junction. The parasitic TFET provides a channel for the supply of emitting electron, while the p-Si/ZnO diode is benefit for impeding the current overloading and prevent the emitters from a catastrophic breakdown. Well repeatable and stable field emission current were obtained from the p-Si/ZnO nano-emitters. High performance nano-emitters was developed using diamond-like-carbon coated p-Si/ZnO tip array (500 × 500), i.e., 178 μA (4.48 mA/cm^2^) at 75.7 MV/m.

Array of well-defined individual field electron emitter is severely desiderated in parallel electron beam lithography^1^,^2^,^3^, vacuum transistors^4^,^5^,^6^, and high frequency amplifier^7^,^8^. The field emission current density and stability, emitter-to-emitter uniformity in an array, and cathode reliability are the key metrics for emitter performance in the majority of these applications. Most of the practical field electron emitter arrays suffer from non-uniform and unreliable emission caused by spatial variation of the emitter profile and effective surface potential barrier. Only a small portion of outstanding emitters in an array contribute field emission. Those outstanding emitters may overload and burn out at a relatively higher current level. *In-situ* integrations of individual ballast resistor^9^, rational designed current limiter^10^,^11^ or field effect transistor^12^,^13^,^14^ in series with emitters are effective to improve their uniformity and reliability. The ballast elements can suppress the excessive emission current and reduce the current fluctuation. It can also improve the emitter-to-emitter uniformity in performance. For example, A. Wisitsora-at *et al*., has fabricated innovative field emitter consisting of an individual nano-diamond tip sitting on a ballast resistor “pole”^9^. The diamond pole ballast resistor provide self-limiting of emission current that improves the total current density as well as the emission current stability. In this strategy, the resistor is a linear load and suppresses the emission in a passive way. L. F. Velásquez-García *et al*., invented an emitter structure of Si tips that are individually ballasted by a high aspect ratio Si column. The Si column utilizes the effect of the velocity saturation of electrons to achieve current-source-like behavior. The technology can be used to implement cathodes capable of uniform and high current emission^10^,^11^. Moreover, there are some other complicated integrated structures that employ active devices such as metal oxide semiconductor field effect transistors (MOSFET)^13^ and thin film transistors (TFT)^14^. The integration of the transistor with the emitter has the featured actively-controlled effect. The supply of the emitted electrons is controlled by the gate voltage of the transistor using the saturation of its conduction channel. Thus, the current stability and uniformity are improved. However, those transistors in series with outstanding emitters may take the risk of drain-source breakdown. It is because the transistors may work in the full saturation region and stand much higher source-drain voltage^15^. Typically, it requires a large transistor channel area and thus the density of the field emitters is limited.

In the present work, we reported an integrated field emitter structure, ZnO nano-emitter individually ballasted by a p-Si/ZnO nano-junction (denoted as p-Si/ZnO nano-emitter). The vertically aligned nano-emitter consists of two segments. The top segment is a ZnO nano-tip (intrinsically lightly n-type doped^16^,^17^) prepared by hydrothermal method. The lower segment is a heavily p-type doped Si nano-pillar. In the field emission measurement, the p-Si/ZnO junction is reversely biased and the field emission current is ballasted by the junction. It is well known that the reversely biased p-n junction would block the current flow^18^,^19^. However, we found that a repeatable and stable field emission current from the p-Si/ZnO nano-emitter array is moderately increased with the rising anode voltage. The field emission current is dramatically higher and the emitters are far more reliable than those of the Si/ZnO nano-emitter arrays with n-n junctions (denoted as n-Si/ZnO nano-emitter). A set of measurements on the electrical and field emission properties of the individual nano-emitters were performed. Combining the experimental evidences with the numerical simulation results, we proposed that the anode voltage not only extracted the electron emission from the emitter, but also induced the inter-band electron tunneling at the surface of the p-Si/ZnO nano-junction. The designed p-Si/ZnO emitter is equivalent to a ZnO nano-emitter individually ballasted by a p-n diode in the interior and a parasitic tunneling field effect transistor (TFET) at the surface of the p-Si/ZnO junction, respectively. The work provides an alternative way to develop field emitters with high current density by utilizing self-modulated electron tunneling at the ballasted p-n junction.

## Results and Discussion

**Figure 1:**
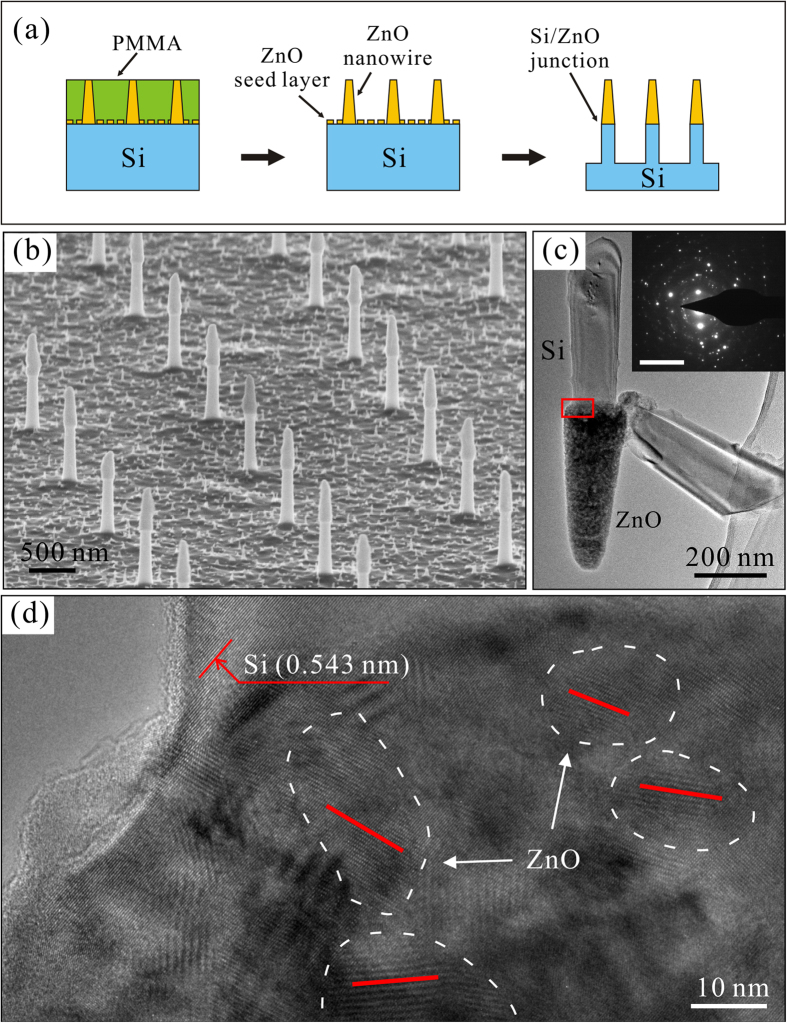
(**a**) The schematic diagram of the fabrication procedure of the integrated p-Si/ZnO nano-electron-emitters. **(b)** The typical SEM image of the p-Si/ZnO nano-emitter array. **(c)** The typical TEM image of a p-Si/ZnO nano-emitter. **(d)** The HRTEM image of the Si/ZnO interface which is in the red rectangle of (**c**), the white dash lines highlight the ZnO nano-crystals, and the red lines indicate the crystal orientation. The corresponding SAED of (**d**) is showed as inset in (**c**) (Scale bar* *=* *10 1/nm).

Figure 1(a) shows the schematic diagram of the fabrication procedure of the integrated p-Si/ZnO nano-electron-emitters. Figure 1(b) is the typical scanning electron microscopy (SEM) image of the p-Si/ZnO nano-emitter array. Figure 1(c) is the typical transmission electron microscopy (TEM) image of a p-Si/ZnO nano-emitter. The length of the top ZnO nano-tip and bottom Si pillar are 500 nm and 650 nm, respectively. The radius of the ZnO nano-tip apex is about 30 nm. The ZnO nano-tip is in well crystalline structure^20^. The high resolution TEM (HRTEM) (Fig. 1(d)) and selected area electron diffraction (the inset of Fig. 1(c)) investigations showing that the Si/ZnO interface is filled with ZnO nano-crystals. The growth of the ZnO nanowire coexists with the etching of the seed layer^21^. Therefore, the nano-crystals are either the pre-deposited seeds or the formed crystal at the beginning of the growth. One of those nano-crystals would dominate the growth to form a crystalline nanowire, while leaving the rest of nano-crystals at the Si/ZnO interface.

**Figure 2:**
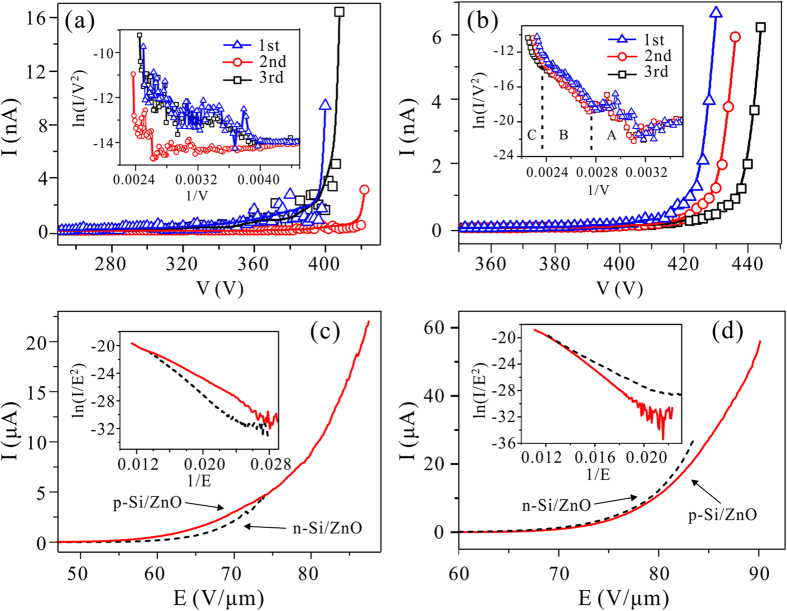
(**a,b**) are the field emission I-V curves and the corresponding F-N plots (the insets) for the individual n-Si/ZnO and p-Si/ZnO nano-emitters, respectively. **(c,d)** are the field emission I-V curves and the corresponding F-N plots (the insets) of the nano-emitter array with 2 μm and 4 μm pitch, respectively.

The n-Si/ZnO nano-emitters are also fabricated for comparative studies. The typical SEM image of the n-Si/ZnO nano-emitters was given in the Supplementary Information, i.e., Figure S1. The p-Si/ZnO and n-Si/ZnO nano-emitters have the similar profile and they are in good uniformity. The field electron emission from the individual nano-emitter and the arrays (500 × 500) were investigated. Figure 2(a) shows the typical emission current *vs* voltage (I-V) curves of a single n-Si/ZnO nano-emitter. The corresponding Fowler–Nordheim (F-N) plots are indicated as insets. The emission current of the single n-Si/ZnO nano-emitter is relatively noisy, which is the typical field emission behavior of the single ZnO nanowire on n-type Si substrates^20^,^22^. The current fluctuation may cause by the surface desorption^23^. Distinctly, the single p-Si/ZnO nano-emitter possesses well reproducible and stable field emission I-V curves (Fig. 2(b)). Higher applied voltage is needed for the p-Si/ZnO nano-emitters to obtain the same emission current, i.e., 425–440 V for 4 nA. Field emission current *vs* electric field (I-E) properties of the nano-emitter arrays respectively with 2-μm-pitch and 4-μm-pitch were investigated. The n-Si/ZnO emitters is likely to fail at an electric field around ~90 MV/m, while that of the p-Si/ZnO emitters is ~ 100 MV/m. Accordingly, the maximum applied fields were carefully controlled to obtain well repeated I-E curves and the typical results are given in Fig. 2(c,d). The I-E curves of the p-Si/ZnO emitters at the low field level showed similar behaviors to that of the n-Si/ZnO emitters. However, the p-Si/ZnO emitters possess much higher capability of field electron emission than that of the n-Si/ZnO emitters. For the p-Si/ZnO emitters with 2-μm-pitch, the emission current is 22 μA at 88 MV/m. Much higher emission current was obtained from the p-Si/ZnO emitters with 4-μm-pitch, i.e., 56 μA at 90 MV/m.

**Figure 3:**
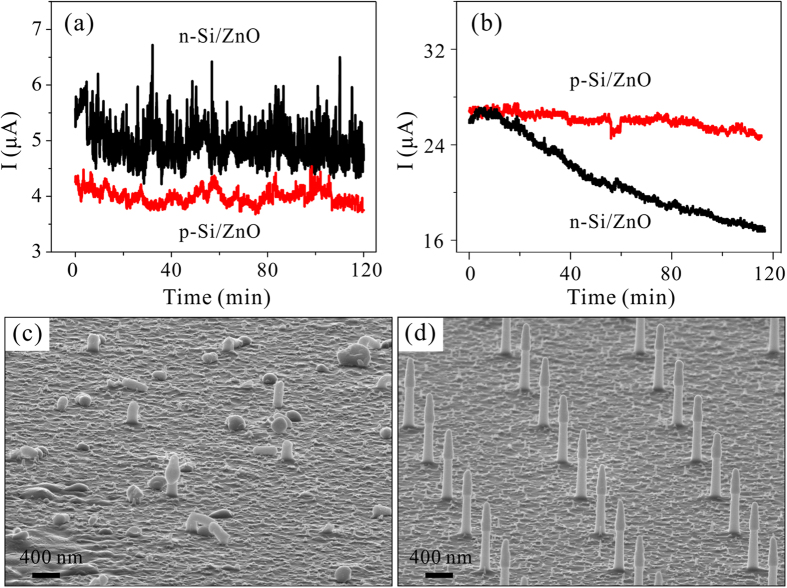
(**a,b**) are the I-t curves of the nano-emitter arrays with 2 μm and 4 μm pitch, respectively. **(c,d)** are the typical SEM images of the n-Si/ZnO and p-Si/ZnO nano-emitter samples after the current stability test, respectively.

The current *vs* time (I-t) properties of the nano-emitter arrays were also measured. The test duration is 2 hours. We defined the current fluctuation as *R = 2σ/I*_*avg*_, where *σ* is the standard deviation and *I*_*avg*_ is the average emission current. As indicated in Fig. 3(a), the current fluctuation of the 2-μm-pitch p-Si/ZnO and n-Si/ZnO emitter arrays are 6.8% and 15.2%, respectively. The n-Si/ZnO emitters showing a slightly current degradation. In Fig. 3(b), the 4-μm-pitch n-Si/ZnO array showed distinct current degradation (black line) and the current dropped by 35.8% in 2 hours. The emission current of the 4-μm-pitch p-Si/ZnO emitters also showed a slight degradation, while the current is relatively stable and the current fluctuation is 7.5%. Figure 3(c,d) are the typical SEM images of the n-Si/ZnO and p-Si/ZnO emitter samples after the current stability tests, respectively. More than 90% emitters in the n-Si/ZnO emitter arrays were broken, while the p-Si/ZnO emitters remained good integrity. The breakdown of the n-Si/ZnO emitters is likely induced by the emission current generated joule heat. Further confirmatory experiments of low voltage parallel electron beam lithography using the Si/ZnO nano-emitter arrays are needed to investigate the emission uniformity of the individual emitters in an array.

**Figure 4:**
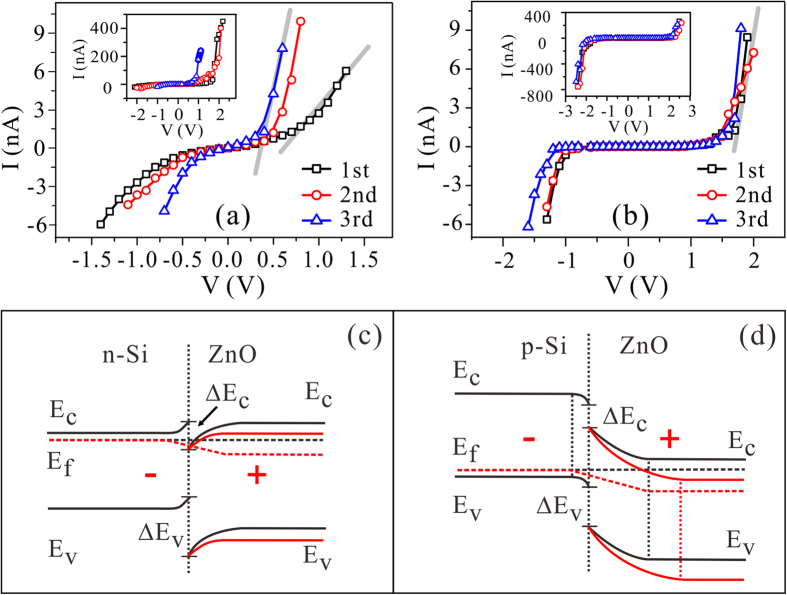
The electric conductance I-V curves for **(a)** the n-Si/ZnO and **(b)** the p-Si/ZnO nano-emitters. The insets are the I-V curves in the larger current range. **(c,d)** are respectively the energy band diagram of the n-Si/ZnO and p-Si/ZnO junctions.

The n-Si/ZnO and p-Si/ZnO nano-emitters are fabricated using the same procedure and the profiles are in good uniformity. The nano-junctions may play a key role for their distinct field emission properties. The investigation on the electric conductance of the individual nano-junctions may provide evidences. Figure 4(a,b) showed the typical conductance I-V curves of the individual n-Si/ZnO and p-Si/ZnO nano-junction, respectively. The corresponding band diagrams of the junctions are also given in Fig. 4(c,d). The dark curves in the figures indicating the band structures of the nano-junctions in thermal equilibrium, while the red curves showing those with applied voltage. The current of the n-Si/ZnO nano-junction gradually rises following the increase of the applied voltage without an obvious threshold. The curves show notable asymmetry in a larger current range (the inset of Fig. 4(a)). The ZnO nanowire prepared by hydrothermal method is lightly n-type doped with carrier concentration of 10^16^~10^18^ cm^−3^ 16,17,24,25,26. The n-Si/ZnO junction is a n-n contact (Fig. 4(c)). The conductance behavior of the n-Si/ZnO junction could be depicted by the thermal electron emission model^27^. Accordingly, the asymmetry of the I-V curves is mainly attributed to the difference of electron density in Si and ZnO. Weak ballasted effect is provided by the n-Si/ZnO junction, result in the noisy field emission and the poor reliability of the n-Si/ZnO emitters. On the other hand, the I-V curves of the p-Si/ZnO nano-junction show the turn-on voltages of −1.0 V and 1.6 V at the forward biased (a negative voltage on the ZnO) and reversely biased (a positive voltage on the ZnO) statuses, respectively. When the p-Si/ZnO nano-junction is forward biased, the current is mainly contributed by electron diffusion, while the threshold is determined by the potential barrier which is estimated to be 0.96 eV for the electron. In the reversely biased status, only “generation current” was recorded when the applied voltage lower than the turn-on voltage. After turn-on, inter-band tunneling dominated the current flow and a remarkable current 0.24 μA was obtained at 2.5 V (inset of Fig. 4(b)). The low turn-on voltage (1.6 V) of the tunneling suggests that it is multi-step tunneling assisted by defect states at the Si/ZnO interface^28^.

**Figure 5:**
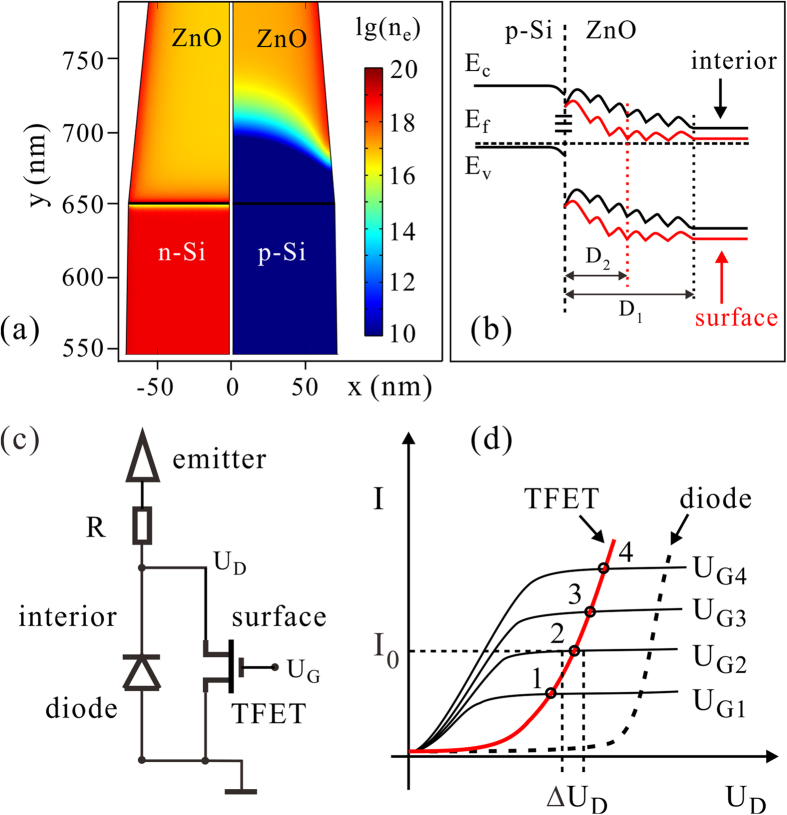
(**a**) The distribution of electron concentration (n_e_) of the n-Si/ZnO nano-junction (left) and p-Si/ZnO nano-junction (right). **(b)** The energy band diagram of the p-Si/ZnO junction taking account of ZnO GBs. The black and red lines show the potential barrier profiles of the interior and surface of the p-Si/ZnO junction, respectively. D_1_ and D_2_ is the width of the depletion region at the interior and surface of the junction. **(c)** The equivalent circuit of the integrated p-Si/ZnO nano-emitter. **(d)** The output characteristics of the parasitic TFET, the I-V curves of the parasitic TFET during field emission (solid red line), and the I-V curve of the interior p-n diode (dashed line).

The inter-band tunneling model provides a clue for understanding the field emission characteristics of the p-Si/ZnO nano-emitters. It is clear that the p-Si/ZnO nano-junction is subjected to different electric field distributions in the field emission measurement and the conductance test. We thus built a two-dimensional axis symmetric model using COMSOL Multi-physics software to qualitatively explore how the field penetration affect the depletion region of the p-Si/ZnO nano-junction during field emission, and further influence the inter-band tunneling. Figure 5(a) shows the simulation results, i.e., the electron distribution of the n-Si/ZnO (left) and p-Si/ZnO (right) nano-junction subjected to the field induced by an anode voltage of 400 V. For the n-Si/ZnO nano-junction, one can see a thin layer of electron accumulation layer beneath the surface of ZnO and there is no depletion region in ZnO. Accordingly, the conductivity of the n-Si/ZnO nano-junction is improved. However, the p-Si/ZnO nano-junction shows a wide depletion region in the ZnO. It is because the Si is heavily p-type doped (1* *×* *10^19^ cm^−3^), while the ZnO is lightly n-type doped (supposed to be 5* *×* *10^16^ cm^−3^). The anode voltage induced the field penetration and formed an electron accumulation layer at the surface of the nano-junction. According to the simulation results, the electron accumulation would reduce the width of the depletion region by a half and enhance the peak electric field to over 5 times compare with those in the center axis of the p-Si/ZnO junction. So the inter-band tunneling current would firstly occur beneath the surface. This is similar to the working principle of the tunneling field effect transistor (TFET)^29^. Due to the fact that the Si/ZnO interface is filled with ZnO nano-crystals. Double Schottky barrier (DSB) will be formed between grain boundaries (GBs) of ZnO at electrostatic and thermal equilibrium^30^. For the ZnO nano-crystals with multi-GBs, the DSBs will overlap one another and form unsmoothed potential profile with small waves^31^. Such a potential profile could still be modulated by the penetrating field^31^. Figure 5(b) schematically represent the band diagram of the p-Si/ZnO interface, taking account of the ZnO nano-crystal GBs. In the ZnO, the energy band (red color) would bent downward due to the penetrated field. Analogous to the simulation results, the depletion region in the interior would be much wider than that at the surface of the junction. Thus the electrons is difficult to transfer from the valence band of Si to the conduction band of ZnO. The penetrated field would reduce the width of the depletion region at the surface of the junction (red lines), induced the band to band tunneling (Zener tunneling) at the junction surface. The defect states at the interface would act as multi-stairs to assist the tunneling. Therefore, it is an important issue to accurately control the density and spatial distribution of the nano-crystal at the p-Si/ZnO interface. However, controlling the nano-crystal density is quite difficult by using the present synthetic process of ZnO nanowires, i.e., hydrothermal method. We proposed that further studies by using molecular beam epitaxy (MBE) or atomic layer deposition (ALD), to do the energy-band engineering at the p-Si/ZnO interface and optimize the performance of the novel nano-emitters.

We propose that the field emission current of the p-Si/ZnO emitter is supplied by the parasitic TFET at the surface of the nano-junction, while the interior p-n junction block the current. Figure 5(c) schematically shows the equivalent circuit. The parasitic TFET at the surface is in parallel with the interior p-n diode. During field emission, the applied anode voltage would extract electron emission from the ZnO apex, which would then poise voltage drop on the p-Si/ZnO junction. This is equivalent to a drain voltage (U_D_) applied on the TFET. The applied anode voltage would also induce field penetration perpendicular to the surface of the p-Si/ZnO junction, which would possess the same effect as a gate voltage (U_G_). Both the U_D_ and U_G_ increase follow the raise of the anode voltage. The field emission current is increased exponentially with the anode voltage, while the electron supply from the parasitic TFET increases in a much slower pace and even gets saturate due to the narrow tunneling channel at the surface. The parasitic TFET is likely to work in the saturation region during field emission. Figure 5(d) schematically reveal the working principle of the p-Si/ZnO nano-emitter. The solid red curve across point 1~4 depicts the working condition of the parasitic TFET during field emission. The four points all locate in the saturation region of the parasitic TFET. Field emission current could be ballasted by the saturation of the parasitic TFET and given stable emission current. With the increasing anode voltage, the current passing through the TFET (finally contributes to field emission) could moderately increase due to the gradually enhancing field penetration. However, if the U_D_ is larger than the breakdown voltage of the reversely biased interior p-n diode, the p-Si/ZnO junction is fully “open”, no ballasted effect can be obtained.

The F-N plots of the single p-Si/ZnO nano-emitters also conform to the parasitic TFET model. As indicated in the inset of Fig. 2(b), the F-N plots can be divided into 3 regions. In region-*A*, the field emission just turns on. The emission current is much lower than the saturation current of the parasitic TFET. The parasitic TFET was working in the liner region, and could not be a good ballast for the emitter. The F-N plots showing obvious fluctuation. The F-N plots in region-*B* coincide well and behave slightly bend down at the medium field, which is similar to the F-N plots in the insets of Fig. 2(c,d). This indicates that the parasitic TFET was working in the saturation region, and ballasted the field emission current from fluctuation. Therefore, higher applied voltage is needed for the p-Si/ZnO nano-emitters to obtain the same emission current than that of the n-Si/ZnO nano-emitter. However, the F-N plots bend upward in region-*C*, which suggests a weaker ballast effect by the parasitic TFET. It is because the parasitic TFET could not supply enough current for field emission, then the voltage drop (U_D_) on the p-Si/ZnO junction would increase. When the U_D_ is comparable to the breakdown voltage (1.6 V) of the reversely biased interior p-n diode, the diode would contribute reverse current. Adequate supply of electron resulted in the up-bend F-N plots. Because each p-Si/ZnO emitter in an array is individually ballasted by a parasitic TFET, they has less probability to become hotspots. More emitters would contribute to the field emission current. The field emission stability and reliability of the p-Si/ZnO array were improved. Thus the p-Si/ZnO array showed higher field emission current and better reliability.

It is also needed to address the difference in field emission performance of the p-Si/ZnO nano-emitter arrays with 2 and 4 μm pitch. According to the earlier literatures^32^,^33^,^34^,^35^, screen effect is a common phenomenon and important issue for the field electron emitter. It has been confirmedly demonstrated that dense arrays possessed stronger field screen effect^32^,^33^,^34^,^35^. Thus, the p-Si/ZnO nano-emitter arrays with 2 μm pitch possess stronger field screen effect than that of the array with 4 μm pitch. Numerical simulation has showed that the local electric field at the apex (E_t_) and the junction surface (E_r_) have decreased by a percentage of 18% and 39%, respectively, when the separation of the emitters reduced from 4 to 2 μm. Although the decrease of E_t_ could reduce the emission current, we believed that the remarkable decrease of the E_r_ may also play a crucial role. Lower E_r_ means lower U_G_ for the parasitic TFET, thus the supply of electrons are limited. Then the voltage drop on the nano-junction would increase, which would then induce the breakdown of the p-Si/ZnO junction. Thus the ballasting effect of the parasitic TFET would lose. Therefore, it is very crucial to ensure sufficient penetrating field strength at the surface of the p-Si/ZnO nano-junction, which means that the p-Si/ZnO nano-junction should expose to the electric field. This is the unique feature of the emitters we studied in the present work^20^,^36^,^37^,^38^. Furthermore, it is very crucial to optimize the tip-to-tip separation in a nano-emitter array to achieve the best field emission performance. Therefore, systematic studies are needed to investigate the effect of emitter density on the emission performance.

**Figure 6:**
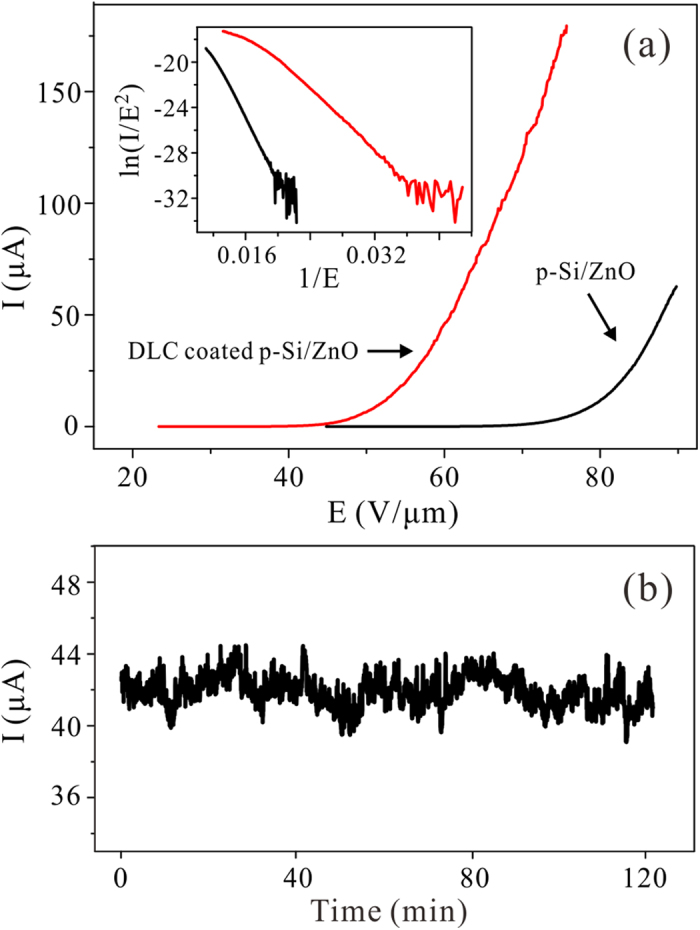
(**a**) The I-E curves and the F-N plots (the inset) of the p-Si/ZnO emitter array with and without the DLC coating, the pith of the array is 4 μm. **(b)** The I-t curve of p-Si/ZnO emitter array with DLC coating.

It has been reported that the high electric field would assist evaporation of oxygen, leaving the zinc rich apex^23^. We believed that it is one of the reasons responsible for the observed current degradation of the 4-μm-pitch p-Si/ZnO emitters (Fig. 2(b)). To further improve the field emission current and the reliability of the p-Si/ZnO array, we employed diamond like carbon (DLC) thin film to cover the p-Si/ZnO emitter surface. Figure 6(a) showed the typical field emission I-E curves and the F-N plots. A field to obtain an emission current of 10 μA is 52.4 MV/m, which is much lower than that of the p-Si/ZnO sample without coating (i.e., 79.5 MV/m). A high current level of 178 μA (4.48 mA/cm^2^) was obtained at 75.7 MV/m. The current is more than 40 times to that of the uncoated p-Si/ZnO emitters at the same applied field (in Fig. 2(b)). Figure 6(b) is the I-t curve of DLC coated p-Si/ZnO emitter array in current stability measurement. The emission current (average value ~43 μA) showed a low fluctuation rate of 4.1% during the 2-hour-test. The enhanced field emission from the DLC coated semiconductor emitters has been comprehensively discussed elsewhere^39^,^40^. Briefly, both the DLC surface and ZnO/DLC interface possess lower potential barrier, which is favorable for field emission. In addition, the well hardness and stronger C-C bond of the DLC would also suppress the surface diffusion and deconstruction of the ZnO apex^40^. The dielectric DLC film is 2 nm in thickness, which may not weaken the field penetration effect. So the p-Si/ZnO nano-junction would ballast the field emission current though the parasitic TFET. As indicated in the inset of Fig. 6(a), the F-N plot bend downward at higher field for DLC coated p-Si/ZnO nano-emitter array.

## Conclusions

High performance and reliable p-Si/ZnO nano-emitters were developed. Both the experimental and simulation results suggested that the anode voltage not only induced the electron emission from the emitter apex but also the inter-band electron tunneling at the surface of the p-Si/ZnO nano-junction. The designed p-Si/ZnO emitter is equivalent to a ZnO nano-emitter individually ballasted by a parasitic TFET at the surface of the p-Si/ZnO junction. The parasitic TFET provides a channel for the supply of emitting electron, while the p-Si/ZnO junction is benefit for impeding the current overloading and prevent the emitters from a catastrophic breakdown. The work provides an alternative way to develop field emitters with self-modulated electron tunneling at the ballasted p-n junction to obtain uniform emitter array with high current density.

## Methods

### Fabrication of the p-Si/ZnO Emitter

The vertically aligned p-Si/ZnO nano-emitter arrays (with 2 and 4 μm pitch) were fabricated using a well-developed fabrication procedure. Heavily boron doped (~1* *×* *10^19^ cm^−3^) <100> Si chip was used as substrates. A layer of zinc (15 nm) was coated on the Si substrate by sputtering and oxidized to form ZnO “seeds” for the growth of ZnO nanowires. The PMMA (polymethyl methacrylate) nano-hole arrays were fabricated on the substrate by electron beam lithography (Raith e-line). Vertically aligned individual ZnO-nano-tips were grown locally in the PMMA nano-hole using the low-temperature hydrothermal method. The ZnO nanowire was grew exceeding the nano hole. The over-growth portion was removed by an ultrasonic bathing, leaving the uniform ZnO nano-tip array in the PMMA nano-holes^20^. Well-defined ZnO nano-tip arrays was obtained by removing the residual PMMA. Followed by etching the seed-layer with oxygen plasma, an anisotropic etch of Si was performed to obtain the vertically aligned p-Si/ZnO nano-emitters. In addition, array of n-Si/ZnO nano-emitter was fabricated for comparative studies following the same procedure. N-type (arsenic-doped; ~1* *×* *10^19^ cm^−3^)* *<* *100> Si chip was used as substrate. Both the p-Si/ZnO and n-Si/ZnO emitters have the similar profile and they are in good uniformity. The nano-emitters were annealed in Ar atmosphere at 400 °C for 2 hours for degasing.

### Morphology and Structure Investigations

The morphology and structural characterization were performed using a scanning electron microscopy (SEM, Zeiss Supra 55) and transmission electron microscopy (TEM, Titan G2 80–300).

### Field Electron Emission and Electrical Tests

The field emission measurements of the nano-emitter arrays (500×500) were performed in an ultra-high vacuum system (4 × 10^−8^ Pa). The typical cathode-anode separation is 35 μm and the anode is an indium tin oxide (ITO) coated glass. A power supply (Keithley 248) and a picoammeter (Keithley 6485) were used for the measurement. The field emission and electric conductance measurements of the individual nano-emitters were carried out using a modified SEM, which is equipped with a precisely manipulated (2 nm per step) anode probe (gold coated tungsten tip). The tip radius of the anode probe is 50 nm and the typical cathode-anode separation is 500 nm. A thin layer of aluminum was coated on the backside of the Si chip for achieving an ohmic-contact between the sample and the holder. In the conductance measurement, a well electric contact was obtained by “melting” the ZnO apex and making it soldered with the probe by current conditioning. A picoammeter (Keithley 6487) was using for both the field emission measurement and the electric contact measurements.

### Simulations

The simulation was performed using Comsol Multiphysics software. We built a 2-dimensional axis symmetric model. The geometry parameters were set according to the nano-emitter samples. The length of the ZnO nanotip and Si pillar is 500 nm and 650 nm, respectively. The diameter of the junction is 120 nm. An anode probe whose radius is 50 nm was placed 500 nm above the ZnO nanotip whose radius is 30 nm. The simulation model would address two coupling processes. Firstly, electric field induced by the anode voltage would cause electron accumulation on the surface of the p-Si/ZnO nano-emitter. Secondly, the redistribution of charge in the p-Si/ZnO emitter would cause the change of the electric field in the vacuum. The two coupling processes would finally reach a balance-state which is determined by the potential continuity boundary condition at the interface between the p-Si/ZnO and vacuum region. More detail of the simulation is presented in the Supplementary Information.

## Additional Information

**How to cite this article**: Cao, T. *et al*. Integrated ZnO Nano-Electron-Emitter with Self-Modulated Parasitic Tunneling Field Effect Transistor at the Surface of the p-Si/ZnO Junction. *Sci. Rep.* **6**, 33983; doi: 10.1038/srep33983 (2016).

## Supplementary Material

Supplementary Information

## References

[b1] Chang T. H. P., Mankos M., Lee K. Y. & Muray L. P. Multiple Electron-Beam Lithography. Microelectronic Engineering 57, 117–135 (2001).

[b2] Cole M. T. . Deterministic Cold Cathode Electron Emission from Carbon Nanofibre Arrays. Scientific Reports 4, 4840 (2014). 24787895 10.1038/srep04840 PMC4007073

[b3] Esashi M., Kojima A., Ikegami N., Miyaguchi H. & Koshida N. Development of Massively Parallel Electron Beam Direct Write Lithography using Active-Matrix Nanocrystalline-Silicon Electron Emitter Arrays. Microsystems & Nanoengineering 1, 15029 (2015).

[b4] Han J. W., Oh J. S. & Meyyappan M. Vacuum Nanoelectronics: Back to the Future? -Gate Insulated Nanoscale Vacuum Channel Transistor. Applied Physics Letters 100, 213505 (2012).

[b5] Wu G. T., Wei X. L., Zhang Z. Y., Chen Q. & Peng L. M. A Graphene-Based Vacuum Transistor with a High ON/OFF Current Ratio. Advanced Functional Materials 25, 5972–5978 (2015).

[b6] Wu G. T., Wei X. L., Gao S., Chen Q. & Peng L. M. Tunable Graphene Micro-Emitters with Fast Temporal Response and Controllable Electron Emission. Nature Communications 7, 11513 (2016). 10.1038/ncomms11513 PMC4866330 27160693

[b7] Teo K. B. K. . Microwave Devices: Carbon Nanotubes as Cold Cathodes. Nature 437, 968–968 (2005). 16222290 10.1038/437968a

[b8] Piot P. . Operation of an Ungated Diamond Field-Emission Array Cathode in A L-Band Radiofrequency Electron Source. Applied Physics Letters 104, 263504 (2014).

[b9] Wisitsora-at A., Hsu S. H., Kang W. P., Davidson J. L. & Tuantranont A. Advanced Nanodiamond Emitter with Pyramidal Tip-on-Pole Structure for Emission Self-Regulation. Journal of Vacuum Science & Technology B 30, 022204 (2012).

[b10] Velásquez-García L. F., Guerrera S. A., Niu Y. & Akinwande A. I. Uniform High-Current Cathodes Using Massive Arrays of Si Field Emitters Individually Controlled by Vertical Si Ungated FETs-Part 2: Device Fabrication and Characterization. Electron Devices, IEEE Transactions on 58, 1783–1791 (2011).

[b11] Guerrera S. A., Velásquez-García L. F. & Akinwande A. I. Scaling of High-Aspect-Ratio Current Limiters for the Individual Ballasting of Large Arrays of Field Emitters. Electron Devices, IEEE Transactions on 59, 2524–2530 (2012).

[b12] Hirano T., Kanemaru S. & Itoh J. A New Metal-Oxide-Semiconductor Field-Effect-Transistor-Structured Si Field Emitter Tip. Japanese Journal of Applied Physics 35, L861–L863 (1996).

[b13] Itoh J., Hirano T. & Kanemaru S. Ultrastable Emission from A Metal-Oxide-Semiconductor Field-Effect Transistor-Structured Si Emitter Tip. Applied Physics Letters 69, 1577–1578 (1996).

[b14] Nagao M. . HfC Field Emitter Array Controlled by Built-in Poly-Si Thin Film Transistor. Journal of Vacuum Science & Technology B 24, 936–939 (2006).

[b15] Yang W. J., She J. C., Deng S. Z. & Xu N. S. Field Emission From a MOSFET-Controlled ZnO-Nanowire Cold Cathode. Electron Devices, IEEE Transactions on 59, 3641–3646 (2012).

[b16] Goldberger J., Sirbuly D. J., Law M. & Yang P. ZnO Nanowire Transistors. The Journal of Physical Chemistry B 109, 9–14 (2005). 16850973 10.1021/jp0452599

[b17] Baxter J. B. & Schmuttenmaer C. A. Conductivity of ZnO Nanowires, Nanoparticles, and Thin Films using Time-Resolved Terahertz Spectroscopy. The Journal of Physical Chemistry B 110, 25229–25239 (2006). 17165967 10.1021/jp064399a

[b18] Kanemaru S., Hirano T., Tanoue H. & Itoh J. Control of Emission Currents from Silicon Field Emitter Arrays using A Built-in MOSFET. Applied Surface Science 111, 218–223 (1997).

[b19] Liu K. X., Chiang C. J. & Heritage J. P. Photoresponse of Gated p-Silicon Field Emitter Array and Correlation with Theoretical Models. Journal of Applied Physics 99, 034502 (2006).

[b20] He H., She J. C., Huang Y. F., Deng S. Z. & Xu N. S. Precisely-Controlled Fabrication of Single ZnO Nanoemitter Arrays and Their Possible Application in Low Energy Parallel Electron Beam Exposure. Nanoscale 4, 2101–2108 (2012). 22333999 10.1039/c2nr11636g

[b21] Liu J., She J. C., Deng S. Z., Chen J. & Xu N. S. Ultrathin Seed-Layer for Tuning Density of ZnO Nanowire Arrays and Their Field Emission Characteristics. The Journal of Physical Chemistry C 112, 11685–11690 (2008).

[b22] She J. C. . Correlation between Resistance and Field Emission Performance of Individual ZnO One-Dimensional Nanostructures. ACS Nano 2, 2015–2022 (2008). 19206446 10.1021/nn800283u

[b23] Semet V., Binh V. T., Pauporté Th., Joulaud L. & Vermersch F. J. Field Emission Behavior of Vertically Aligned ZnO Nanowire Planar Cathodes. Journal of Applied Physics 109, 054301 (2011).

[b24] Yang X. . Nitrogen-Doped ZnO Nanowire Arrays for Photoelectrochemical Water Splitting. Nano Lett. 9, 2331–2336 (2009). 19449878 10.1021/nl900772q

[b25] Liu Y., Zhang Z. Y., Wei X. L., Li Q. & Peng L. M. Simultaneous Electrical and Thermoelectric Parameter Retrieval via Two Terminal Current–Voltage Measurements on Individual ZnO Nanowires. Adv. Funct. Mater. 21, 3900–3906 (2011).

[b26] Ginting R. T. . A Simple Approach Low-Temperature Solution Process for Preparation of Bismuth-Doped ZnO Nanorods and Its Application in Hybrid Solar Cells. J. Phys. Chem. C 120, 771–780 (2016).

[b27] Sze S. M. & Ng K. K. Physics of Semiconductor Devices . Ch. 3, 154–158 (John wiley & sons, 2006).

[b28] Riben A. R. & Feucht D. L. Electrical Transport in nGe-pGaAs Heterojunctions. International Journal of Electronics 20, 583–599 (1966).

[b29] Ionescu A. M. & Riel H. Tunnel Field-Effect Transistors As Energy-Efficient Electronic Switches. Nature 479, 329–337 (2011). 22094693 10.1038/nature10679

[b30] Blatter G. & Greuter F. Carrier Transport Through Grain Boundaries in Semiconductors. Physical Review B 33, 3952 (1986). 10.1103/physrevb.33.3952 9938810

[b31] Hossain F. M. . Modeling and Simulation of Polycrystalline ZnO Thin-Film Transistors. Journal of Applied Physics 94, 7768–7777 (2003).

[b32] Yana X. B., Taya B. K. & Miele P. Field Emission from Ordered Carbon Nanotube-ZnO Heterojunction Arrays. Carbon 46, 753–758 (2008).

[b33] Harris J. R., Jensen K. L. & Shiffler D. A. Dependence of Optimal Spacing on Applied Field in Ungated Field Emitter Arrays. AIP Advances 5, 087182 (2015).

[b34] Pan N. . Tip-Morphology-Dependent Field Emission from ZnO Nanorod Arrays. Nanotechnology 21, 225707 (2010). 20453277 10.1088/0957-4484/21/22/225707

[b35] Zeng H. B. . Template Deformation-Tailored ZnO Nanorod/Nanowire Arrays: Full Growth Control and Optimization of Field-Emission. Adv. Funct. Mater. 19, 3165–3172 (2009).

[b36] Kim Y. J., Lee C. H., Hong Y. J. & Yi G. C. Controlled Selective Growth of ZnO Nanorod and Microrod Arrays on Si Substrates by a Wet Chemical Method. Appl. Phys. Lett. 89, 163128 (2006).

[b37] Ahsanulhaq Q., Kim J. H. & Hahn Y. B. Controlled Selective Growth of ZnO Nanorod Arrays and Their Field Emission Properties. Nanotechnology 18, 485307 (2007).

[b38] Xu S. . Patterned Growth of Vertically Aligned ZnO Nanowire Arrays on Inorganic Substrates at Low Temperature without Catalyst. J. Am. Chem. Soc. 130, 14958 (2008). 18921981 10.1021/ja806952j

[b39] Xu N. S., She J. C., Huq S. E., Chen J. & Deng S. Z. Enhancing Electron Emission from Silicon Tip Arrays by Using Thin Amorphous Diamond Coating. Applied Physics Letters 73, 3668–3670 (1998).

[b40] Huang Y. F. . Field-Induced Crystalline-to-Amorphous Phase Transformation on the Si Nano-Apex and the Achieving of Highly Reliable Si Nano-Cathodes. Scientific Reports 5, 10631 (2015). 25994377 10.1038/srep10631 PMC4440211

