# Supplementary material for: Integrated ZnO Nano-Electron-Emitter with Self-Modulated Parasitic Tunneling Field Effect Transistor at the Surface of the p-Si/ZnO Junction
Source: Sci Rep. 2016 Sep 22;6:33983. doi: 10.1038/srep33983 (PMC5032023; doi:10.1038/srep33983)
Supplement: Supplementary Information [file srep33983-s1.pdf]

Supplementary Information

Integrated ZnO Nano-Electron-Emitter with Self-Modulated Parasitic Tunneling Field Effect Transistor at the Surface of the p-Si/ZnO Junction

Tao Cao [a)], Laitang Luo [a)], Yifeng Huang [a)], Bing Ye [a)], Juncong She*[, a), b)], Shaozhi Deng [a)], Jun Chen [a)], Ningsheng Xu [a)]

[a)] State Key Laboratory of Optoelectronic Materials and Technologies, Guangdong Province Key Laboratory of Display Material and Technology, School of Electronics and Information Technology, Sun Yat-sen University, Guangzhou 510275, People's Republic of China

[b)] Sun Yat-sen University-Carnegie Mellon University (SYSU-CMU) Shunde International Joint Research Institute, Shunde 528300, People's Republic of China

*Address correspondence to shejc@mail.sysu.edu.cn

1. Equation and boundary condition of the simulation

In the domains of the emitter, the relation between the carrier concentration and potential distribution can be determined by Poison equation,

$$\nabla \cdot (\varepsilon_r \nabla \varphi_{semi}) = q(n - p + N_A^- - N_D^+)$$

Where φ_{semi} is the potential in the nanowire domains. ε_r is the relative permittivity for Si or ZnO. q is charge of one electron. n, p, N_A^-, N_D^+ is concentration for electrons, holes, ionized acceptors and ionized donors.

The current continuity equations depicts the carrier redistribution in the applied electric field,

$$\frac{1}{q}\nabla \cdot J_n = -U_n$$
$$\frac{1}{q}\nabla \cdot J_p = -U_p$$

Where J_n, J_p is current of electrons and holes. U_n, U_p is recombination rate for electrons and holes and here they are set to 0.

In the vacuum domain (other space except for the nanowire domain) the charge density is 0, thus the potential field can be depicted by Laplace equation,

$$\nabla \cdot (\nabla \varphi_{es}) = 0$$

The equations for boundary conditions include,

$$\varphi_{probe} = U_a$$

$$\varphi_{btm} = 0$$

$$\varphi_{semi.bdr} = \varphi_{es.bdr}$$

$$\varepsilon_r \frac{\partial \varphi_{semi.bdr}}{\partial \boldsymbol{n}} = \varepsilon_0 \frac{\partial \varphi_{es.bdr}}{\partial \boldsymbol{n}}$$

Where φ_{es} is the potential in the vacuum domain. φ_{probe} is the potential of the

anode probe. φ_{btm} is the potential of the bottom line of the calculation area. $\varphi_{semi.bdr}$ and $\varphi_{es.bdr}$ is the potential of nanowire side and vacuum side at the interface of the nanowire and vacuum. ε_0 is the electric permittivity of vacuum. **n** is the normal vector of the surface of nanowire domains.

2. The Typical SEM Images of the n-Si/ZnO Emitters

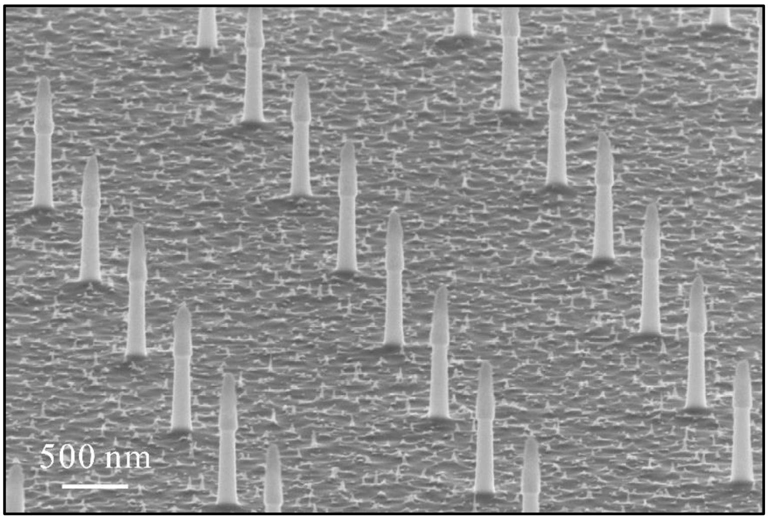

Figure S1: The typical SEM images of the n-Si/ZnO. The n-Si/ZnO and p-Si/ZnO nano-emitters have the similar profile and they are in good uniformity.
